# Characterization of the canine CD20 as a therapeutic target for comparative passive immunotherapy

**DOI:** 10.1038/s41598-022-06549-1

**Published:** 2022-02-17

**Authors:** Joana N. R. Dias, André Almeida, Ana S. André, Sandra I. Aguiar, Pedro Bule, Sara Nogueira, Soraia S. Oliveira, Belmira Carrapiço, Solange Gil, Luís Tavares, Frederico Aires-da-Silva

**Affiliations:** 1 CIISA-Centro de Investigação Interdisciplinar em Sanidade Animal, Faculdade de Medicina Veterinária, Universidade de Lisboa, 1300-477 Lisbon, Portugal; 2 Technophage SA, Avenida Prof. Egas Moniz, Edifício Egas Moniz, 1649-028 Lisbon, Portugal

**Keywords:** B-cell lymphoma, Oncology

## Abstract

Anti-CD20 therapies have revolutionized the treatment of B-cell malignancies. Despite these advances, relapsed and refractory disease remains a major treatment challenge. The optimization of CD20-targeted immunotherapies is considered a promising strategy to improve current therapies. However, research has been limited by the scarcity of preclinical models that recapitulate the complex interaction between the immune system and cancers. The addition of the canine lymphoma (cNHL) model in the development of anti-CD20 therapies may provide a clinically relevant approach for the translation of improved immunotherapies. Still, an anti-CD20 therapy for cNHL has not been established stressing the need of a comprehensive target characterization. Herein, we performed an in-depth characterization on canine CD20 mRNA transcript and protein expression in a cNHL biobank and demonstrated a canine CD20 overexpression in B-cell lymphoma samples. Moreover, CD20 gene sequencing analysis identified six amino acid differences in patient samples (C77Y, L147F, I159M, L198V, A201T and G273E). Finally, we reported the use of a novel strategy for the generation of anti-CD20 mAbs, with human and canine cross-reactivity, by exploring our rabbit derived single-domain antibody platform. Overall, these results support the rationale of using CD20 as a target for veterinary settings and the development of novel therapeutics and immunodiagnostics.

## Introduction

Anticancer immunotherapies are rapidly transforming the field of oncology, providing unprecedented and long-lasting clinical efficacies in a wide range of malignancies^1,2^. Antibody-based therapy benefits have particularly impacted hematological cancer treatments. Rituximab anti-cancer therapy poses by far as one of the most successful examples. Since its approval in 1997 by the Food and Drug Administration (FDA), anti-CD20 therapy has revolutionized the treatment of B-cell hematological malignancies, namely for indolent and aggressive subtypes of B-cell non-Hodgkin (NHL) and chronic lymphocytic leukemia (CLL), reducing mortality and improving the overall prognosis of patients with acceptable toxicity^3,4^. The CD20 is a 33–37-kDa non-glycosylated transmembrane phosphoprotein member of the membrane-spanning 4-A (MS4A) family that is found on all mature B-cells and that typically presents a constitutive and constant expression. Importantly, CD20 is found in 95% of B-cell malignancies representing an appealing therapeutic target. By engaging Fc receptors on immune effector cells, rituximab activates complement-dependent cytotoxicity (CDC) and antibody-dependent cell-mediated cytotoxicity/phagocytosis (ADCC/ADCP), while eliciting direct cytotoxic and pro-apoptotic effects^4,5^. Due to its impressive clinical efficacy, rituximab success has prompted the development of a new generation of therapeutic anti-CD20 monoclonal antibodies (mAbs) (e.g., obinutuzumab, ofatumumab, veltuzumab, and ocrelizumab). However, their efficacy and safety compared with rituximab are still controversial and rituximab maintains a leading position in standard care^6^. Even though alternative mAbs targeting other tumor antigens have been ensued, the clinical responses achieved with these antibody-based therapies have only been modest and CD20-targeting mAbs continue to represent more than 30% of all therapeutic mAbs for hematological malignancies^7^. Beyond malignant disease, anti-CD20 therapies that result in B-cell compartment depletion have also gained relevance in the treatment of inflammatory and autoimmune diseases^8^.

Despite the unprecedented clinical success of rituximab and other anti-CD20 mAbs, a substantial fraction of patients relapse or are resistance to treatment, highlighting the need for more effective therapies^4,6^. The optimization of immunotherapy CD20 targeted drugs is considered one of the most promising strategies to improve the clinical efficacy and safety of current therapies. Nonetheless, anti-cancer CD20 targeted therapy research has been limited in part by the scarcity of preclinical models that fully mimic the heterogeneity and complexity of the in vivo interaction between the human immune system and cancers^4^.

Naturally occurring NHL in dogs share several clinical, pathological, immunologic, molecular and therapeutic resemblances with its human counterpart, that are difficult to replicate in conventional preclinical models, making the dog a promising animal model to explore and validate novel therapeutic options^9–11^. Canine lymphoma (cNHL) represents an heterogeneous group of malignancies that are among the most common neoplasias of dogs. Although lymphoma in dogs can display a great variety of clinical presentations and histological subtypes, most animals present with generalized lymphadenopathy (multicentric form) and intermediate to high-grade subtypes, more frequently of B-cell origin^12^. Cyclophosphamide, doxorubicin, vincristine and prednisone (CHOP) chemotherapy regimen is at the core of cNHL treatment, however this treatment modality is rarely curative and most dogs relapse with lethal, drug-resistant disease^13–15^. Therefore, there is also an pressing need in veterinary medicine to develop novel treatment approaches for intractable disease^13^. Hence, motivated by the impressive success attained by immunotherapies in human NHL, comparative oncology has invested on the development of similar immunotherapeutic approaches for dogs. Although several efforts have been pursued, an antibody-based therapy for cNHL has not yet been established. Immunohistochemistry using antibodies that bind the CD20 intracellular domains confirmed CD20 expression in cNHL tissue samples^16,17^. However, rituximab and other anti-human and anti-mouse mAbs specific to the human CD20 extracellular domains did not bind to canine CD20, albeit the proposed binding epitopes are conserved between human and canine CD20^18^. This highlighted that the development of similar passive immunotherapy strategies for canine cancer patients requires the canine speciation of antibodies. A few anti-canine CD20 mAbs, clone 6C8^19^, clone 1E4^20^, NCD1.2^21^, Blontuvetmab (Aratana) and 4E1-7-B_f^22^ have been previously reported. However, to present none of these mAbs has peer reviewed clinical data evaluating its efficacy and safety in B-cell lymphoma patients.

The hurdles encountered by these efforts point out that the development of an effective anti-CD20 passive immunotherapy for cNHL requires a comprehensive target characterization and a better understanding of antibody effector functions of the canine immune system. Within this context and to fulfill these gaps, we conducted for the first time an in-depth investigation on canine CD20 by evaluating the CD20 mRNA transcript and protein expression as well as a CD20 gene sequencing analysis in a canine multicentric lymphoma biobank. Moreover, we described the use of a new approach for the development of recombinant anti-CD20 mAbs that explores our rabbit derived single-domain antibody (sdAbs) platform. Through this approach we intend to contribute for the development of more effective immunotherapies and immunodiagnostics for cNHL, while adding value to comparative oncology.

## Results

### Characterization of canine CD20 expression in a canine multicentric lymphoma biobank

**Figure 1:**
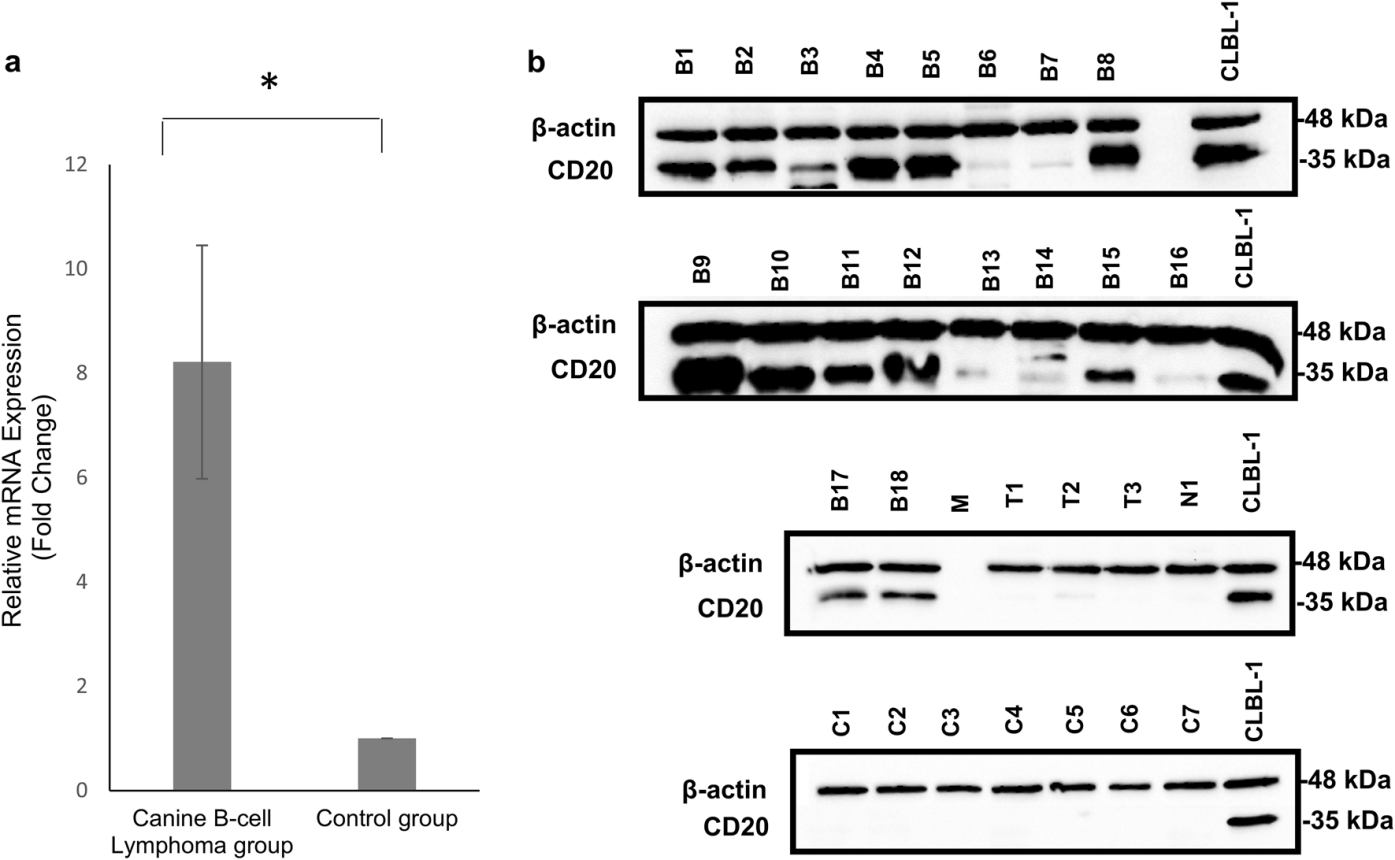
Canine CD20 expression characterization validates the membrane overexpression of CD20 in B-cell lymphoma. (**a**) Relative CD20 mRNA expression in cNHL tumor tissue. Results are expressed as a fold difference between mean ± SEM of mRNA expression level in cNHL affected lymph nodes samples and a control group with healthy donors. *p < 0.05. (**b**) Representative blots of CD20 WB analysis. Total protein was extracted from cNHL and healthy donor lymph nodes and CD20 expression was assessed by western blot using an anti-CD20 polyclonal antibody (CD20). CLBL-1 cell line was used as a control and loading was controlled with an anti-β-actin monoclonal antibody (β-actin). (B1–B18 represent biobank B-cell lymphoma samples; T1–T3 represent biobank T-cell lymphoma samples; N1 represent a biobank non-B non-T cell lymphoma sample; C1–C7 represent healthy donor samples.

CD20 expression has been demonstrated to be heterogeneous between and within different subtypes of the human B-NHL and has been considered a predictor of response to rituximab based treatment in patients with B-cell lymphomas^23^. As such, a comprehensive analysis of the expression of CD20 in both healthy and B-cell lymphoma diagnosed dogs might also have a critical role in the development of a similar canine anti-CD20 immunotherapy. Therefore, to further characterize CD20 gene and protein expression in cNHL, relative quantification of CD20 mRNA transcript expression by qRT-PCR and CD20 protein analysis by western blot (WB) was performed on 22 patient samples of affected lymph nodes from a canine multicentric lymphoma biobank previously established by our group^24^. Briefly, our biobank was constructed from patients with canine multicentric lymphoma followed at the oncology unit at the Teaching Hospital of the Faculty of Veterinary Medicine, University of Lisbon, where clinical evaluations were conducted, and histopathological and immunochemistry analysis were performed^24^. The biobank contains samples from B-cell lymphoma, T-cell lymphoma and non-B and non-T cell lymphoma^24^ (Supplementary Table S1). As shown in Fig. 1a, the qRT-PCR results demonstrated that the mRNA expression levels of canine CD20 were significantly higher in B-cell lymphoma samples when compared with samples derived from healthy lymph nodes and non-B-cell lymphoma, including T-cell and non-B and non-T cell lymphoma samples (p < 0.05). This increment was quantified as 8.2 ± 2.23 times fold, validating the CD20 mRNA overexpression in canine B-cell lymphoma samples derived from the biobank (Fig. 1a). Regarding CD20 protein expression by WB analysis, all B-cell lymphoma samples demonstrated a CD20-positive phenotype. Moreover, CD20 expression was found to be heterogeneous on B-cell lymphoma samples. In contrast, samples derived from T-cell and non-B and non-T cell lymphoma was not detectable or presented a scanty CD20 expression (Fig. 1b). In samples derived from the healthy lymph nodes the CD20 expression was not detectable. The data obtained in the WB analysis corroborated the qRT-PCR data, demonstrating a canine CD20 overexpression in B-cell lymphoma samples and thus supporting the rationale of using the CD20 as a promising B-cell lymphoma immunotherapy target for the veterinary setting.

### Sequence analysis of the canine MS4A1 (CD20) gene in a canine multicentric lymphoma biobank

**Figure 2:**
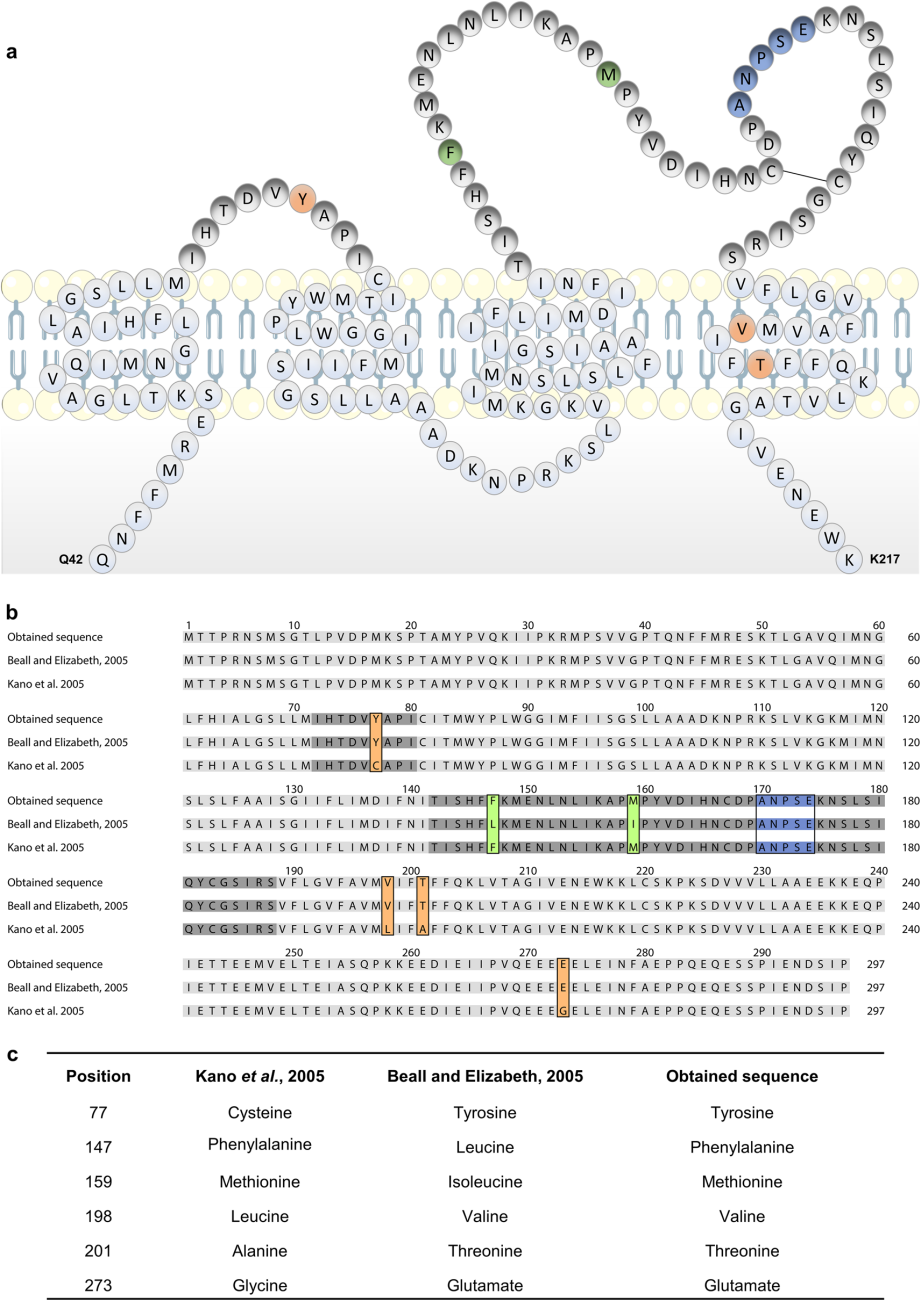
Canine lymphoma biobank sequence shows coding differences from previous published sequences. (**a**) Schematic representation of the amino acid sequence described in this study, coding for the transmembrane portion of the canine CD20 protein according to Rougé et al.^45^. (**b**) Alignment of CD20 sequence described in the present study and compared with both previous published canine CD20 sequence^17,27^. Colored amino acids represent substitutions relative to the previous published sequences and rituximab epitope (Orange-Kano et al., Green-Beall and Elizabeth, Blue—rituximab epitope ANPSE). (**c**) Table detailing coding differences leading to amino acid substitutions described in the present study compared with previous published sequences.

The first canine CD20 gene sequence analysis was reported in 2005 by Kano et al.^17^. In this study the authors demonstrated that the full-length of the cDNA sequence of canine CD20 consisted in 1239 bp encoding 297 amino acids. This amino acid sequence exhibited 73 and 68% similarities with human and mouse CD20 sequences (GenBank accession number AAA35581.1 and AAA37394.1, respectively), suggesting that the CD20 receptor might play a role in the immune system similar to other mammals^17^. Moreover, the canine CD20 was predicted to contain four membranes spanning domains, three intracellular domains including the N- and C-terminal domains and two extracellular domains as the human CD20^25,26^. A second canine CD20 sequence was further reported by Beall and Elizabeth^27^ (GenBank accession number ACS04289.1). This canine CD20 sequence was reported to encode 297 amino acids with 6 sequence differences (C77Y, F147L, M159I, L198V, A201T, G273E) when compared with the CD20 sequence previously reported by Kano et al*.*^17^. Potential differences in the coding sequence of the canine CD20 might have a key role in the development of CD20 therapeutic mAbs and explain the absence of cross-reaction between species, especially if these genetic alterations occur in the specific binding domains. Therefore, in the present study aiming to contribute with more data to characterize the canine CD20, we performed CD20 sequencing analysis of samples derived from our canine multicentric lymphoma biobank and of lymph nodes and PBMCs of healthy donors. The obtained sequencing data for all samples presented, as expected, a CD20 sequence that encoded a protein with 297 amino acids. However, this canine CD20 sequence diverged by 6 sequence differences (Fig. 2) when compared with the two previously reported sequences^17,27^. Importantly, the amino acid sequence differences observed are a combination of the differences between the two reported sequences^17,27^. Indeed, if we compare the CD20 sequence deposited in the GenBank by Kano et al. with our sequence we can observe 4 amino acid sequence differences in positions 77, 198, 201 and 273 corresponding to the substitutions C77Y, L198V, A201T and G273E in the canine CD20 protein. In addition, if we compare the canine CD20 sequence deposited by Beall and Elizabeth with our sequence we can observe 2 amino acid sequence differences in positions 147 and 159 which lead to the substitutions L147F, I159M. As shown in Fig. 2, the amino acid differences identified are positioned in the CD20 cytoplasmic and transmembrane portions (L198V, A201T and G273E) and importantly in the two extracellular loops (C77Y, L147F, I159M) that are exposed and are potential epitopes of therapeutic and diagnostic antibodies for B-cell lymphoma. Thus, our study contributed with a better understanding of canine CD20 gene sequence for the development of anti-CD20 mAbs for veterinary use.

### Development of sdAbs against canine CD20

#### Generation and screening of high-tittered antisera against canine CD20

**Figure 3:**
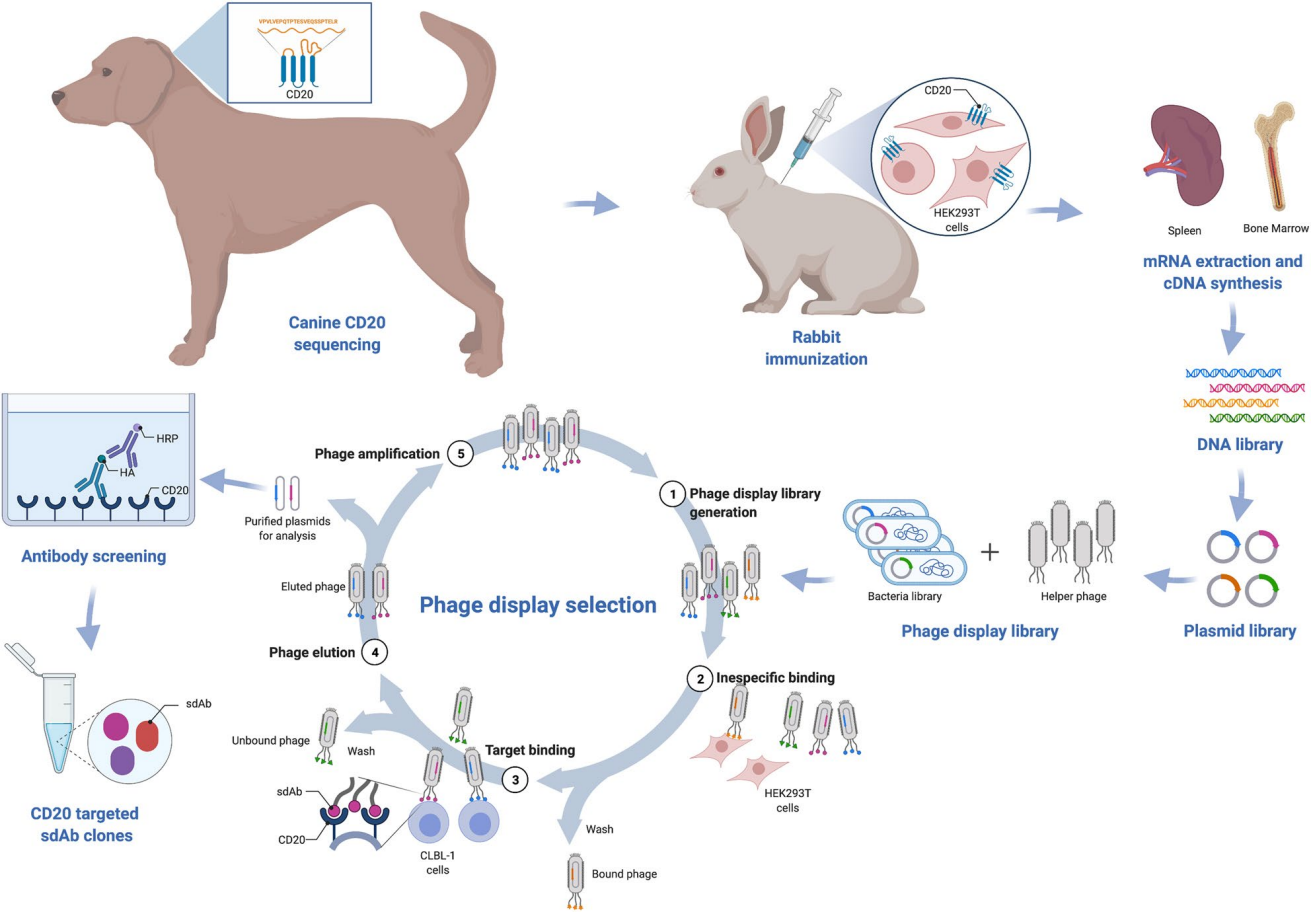
Schematic illustration of the adopted strategy for the development of rabbit derived sdAbs against canine CD20. An anti-CD20 sdAbs library was constructed from an immunized rabbit with HEK 293 T cells transfected with canine CD20 vector. Selection of specific sdAbs for the canine CD20 receptor was performed using a subtractive phage display screening of the library on native-like receptor-expressed on cells. For further selection of the best candidates, clone binding and expression activity were further tested and confirmed by ELISA.

**Figure 4:**
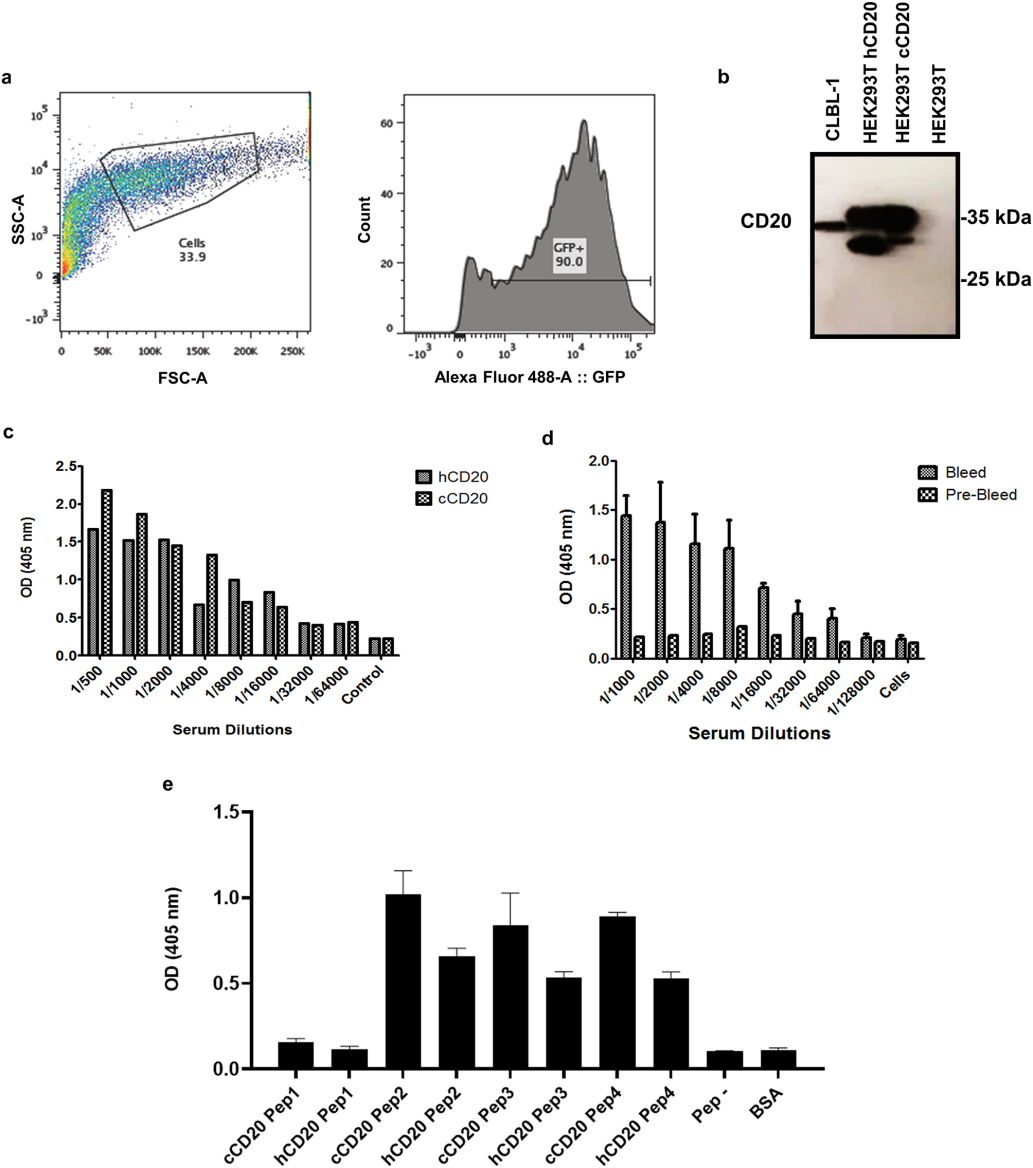
Generation and screening of high-titered antisera against canine CD20. (**a**) Generation of a HEK 293 T cell line with transient expression of canine CD20. Representative histogram of the construction of a HEK 293 T cell line with transient expression of canine CD20, representing GFP expression analysis 48 h after cells transfection with FuGENE^®^ HD and pFUGW vector with CD20 and GFP reporters. (**b**) Representative blots of the immunoblotting analysis, confirming CD20 expression of HEK 293T^cCD20+GFP+^ and 293T^hCD20+GFP+^ cell lines. Total protein was extracted from 293T^cCD20+GFP+^ and 293T^hCD20+GFP+^ cell lines and CD20 expression was assessed by WB using an anti-CD20 polyclonal antibody (CD20). CLBL-1 cell line was used as a positive control and HEK 293 T cell line as a negative control. (**c**) Titration and binding activity of sera antibodies of the rabbit pre bleed and final bleed to 293T^cCD20+GFP+^ and 293T^hCD20+GFP+^ cell lines. (**d**) Titration and binding activity of sera antibodies of the rabbit pre bleed and final bleed to CLBL-1 cell line. Antisera from the immunized rabbit was analyzed for binding by ELISA using HRP-conjugated goat anti-rabbit Fc polyclonal antibody as secondary antibody. Data were obtained by absorbance measurement at 405 nm. Results are expressed as mean ± SEM. (**e**) To perform epitope mapping, rabbit serum was tested for binding to a set of overlapping synthetic CD20 canine and human peptides (Supplementary Table S1) covering the two entire external domains (ED1 and ED2). Results were measured by optical density at 405 nm. Results are expressed as mean ± SEM.

Collected data from the target characterization studies revealed that the canine CD20 receptor show potential as an immune target for diagnostic and therapeutic use in veterinary settings. Despite the increasing research in cNHL, there is no anti-CD20 antibody-based therapy clinically available for cNHL to date. To develop an effective CD20 targeted antibody-based therapy, we explored our rabbit derived sdAbs platform to develop novel recombinant anti-CD20 antibodies scaffolds against the coding sequence described herein. This research strategy is depicted in Fig. 3. Briefly, a female New Zealand white rabbit was immunized with HEK 293 T cells transfected with a canine CD20 vector to generate a highly specific serum against canine CD20. For that purpose, the HEK 293 T cell line was transfected with a pFUGW vector encoding canine CD20 and a GFP reporter, as described in the material and methods section. While flow cytometry analysis confirmed GFP expression in HEK 293T^cCD20^ cell line, immunoblotting analysis confirmed the CD20 expression (Fig. 4a,b). In parallel, HEK 293T^hCD20^ was simultaneously generated for cross-reactivity analysis purposes. To monitor the triggered rabbit immune response against CD20, antibody titers and specificity were evaluated by enzyme-linked immunosorbent assay (ELISA). As shown in Fig. 4c, cell ELISA assays showed that the final bleed serum recognized both HEK 293T^cCD20^ and HEK 293T^hCD20^ cells contrarily to pre-bleed serum, confirming cross-reactivity against human CD20. Moreover, these data demonstrated that immunizations resulted in a strong immune response with a high serum titer (1:60,000), as was expected for rabbit immunizations with cells. Similar results were obtained in the ELISA assay using CLBL-1, a canine B-cell lymphoma cell line^28^, that express canine CD20 in its native form (Fig. 4d).

Next, to evaluate the binding epitope profile by the rabbit sera, we synthetized a set of 4 overlapping synthetic peptides covering the two extracellular canine and human CD20 loops. Interactions between the rabbit serum and each synthetic peptide was assessed by ELISA. As shown in Fig. 4e, results demonstrated that the anti-CD20 rabbit serum presented the highest binding against the second CD20 extracellular loop. These results are consistent with currently available data about anti-human CD20 antibodies, reporting that most epitopes involved in antibody recognition are located within the second and larger extracellular loop. Moreover, the serum shown a cross-specificity against the second human CD20 extracellular loop and no binding was shown against an irrelevant peptide. Altogether, these results allowed to confirm a high antisera titer against canine and human CD20, indicating that cell immunizations triggered a specific B-cell reaction against the CD20 receptor, which will guarantee a high-quality antibody gene library. As a result, the immunized rabbit was sacrificed, and the bone marrow and the spleen were extracted for total RNA preparation and cDNA synthesis.

#### Construction of a rabbit anti-CD20 sdAb library and phage display selection of lead candidates

**Figure 5:**
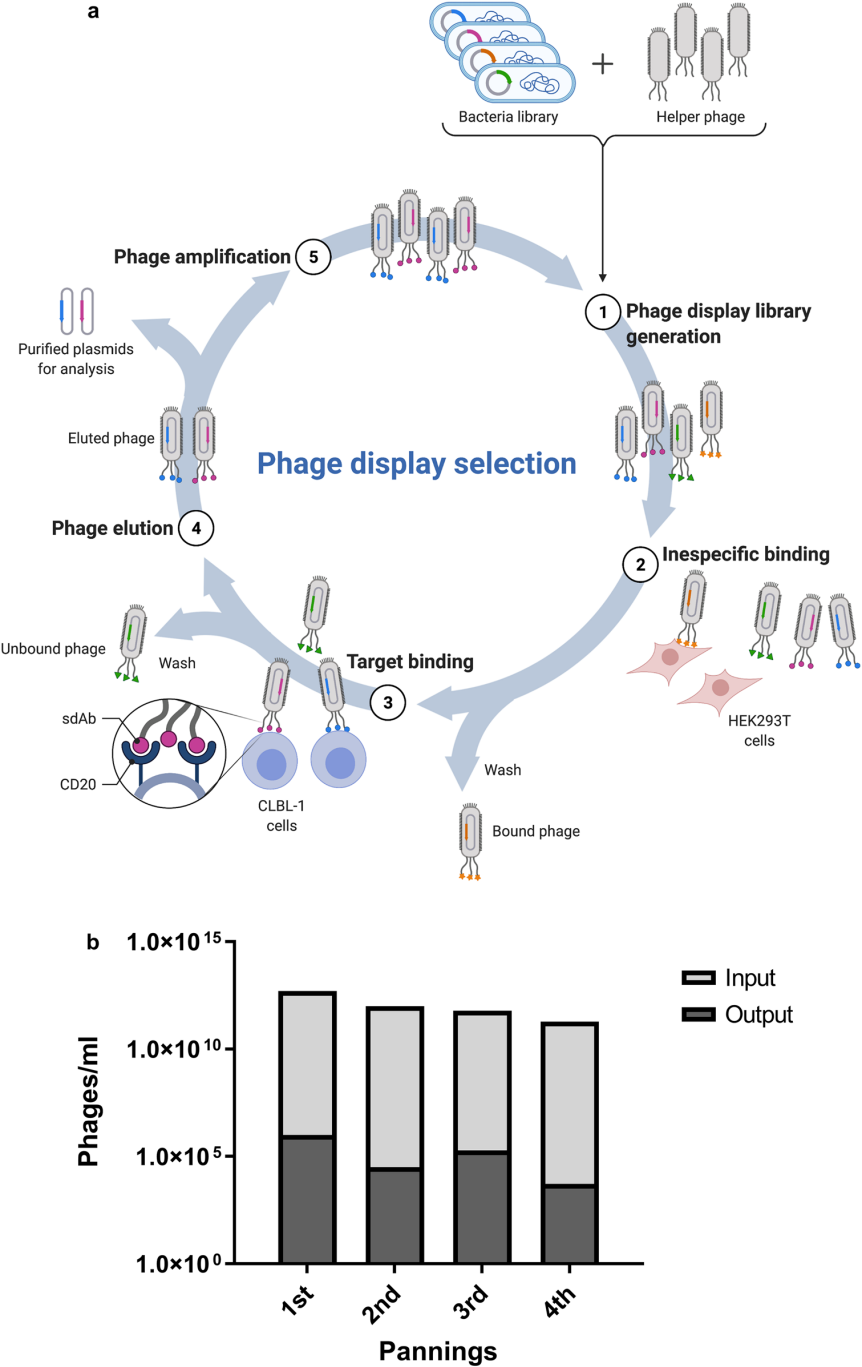
Selection of specific sdAbs for the canine CD20 receptor were performed using a subtractive cell phage display. (**a**) Schematic illustration of the cell phage display screening protocol based on the previously Carlos Barbas studies^34^, in which it is reported a novel whole-cell selection protocol with negative and positive selection steps (subtractive phage display) designed to remove antibodies reacting with common antigens. (**b**) Biopanning screening results obtained for the sdAb V_L_ selection by phage display.

**Figure 6:**
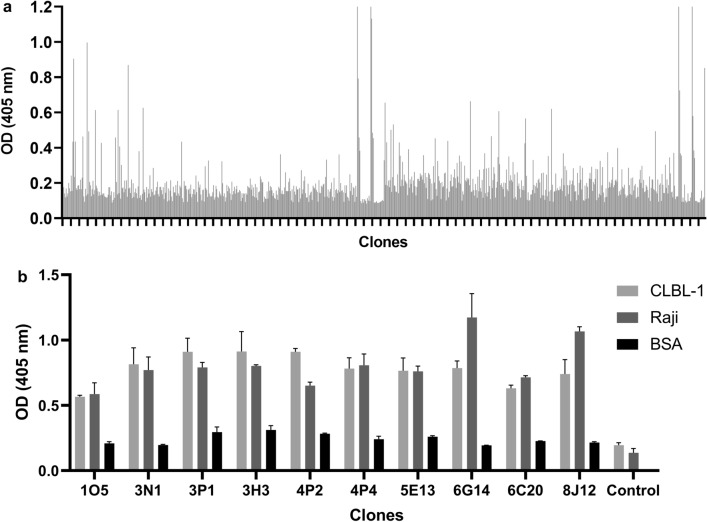
Screening and selection of the lead anti-CD20 sdAbs candidates. (**a**) High throughput binding activity screening of a total of 528 sdAb V_L_ clones. Binding activity of the best V_L_s candidates selected by an high throughput screening were assessed by ELISA. (**b**) Clones binding activity was tested by ELISA using CLBL-1 cell extract. To evaluate cross-reactivity against human CD20, the same experiment was carried out using Raji cell extract. HRP-conjugated anti-HA mAb was used as secondary antibody. BL21 extracts were used as negatives controls. Results were measured by optical density at 405 nm. Results are expressed as mean ± SEM.

To select the most promising antibodies for canine CD20 targeting, a rabbit sdAb library was constructed using the cDNA synthesized from total RNA extracted from spleen and bone marrow cells. For that purpose, specific oligonucleotide primers covering all known variable rabbit antibody family sequences were used to amplify sdAbs derived from light chain variable regions (V_L_) gene segments^29^. The construction of a V_L_ library was chosen due to the high diversity presented by the rabbit V_L_ repertoire and stability when compared to the heavy chain variable regions (V_H_) repertoire^29^. Furthermore, the rabbit V_L_ repertoire possess other advantages such as its higher diversity and larger complementarity-determining region (CDR)-L3 loop length, superior to the mouse and human immunological repertoire, which enables the generation of large libraries against difficult epitopes^30–33^. The recombinant phagemid pComb3X containing the V_L_ sdAb genes was transformed into *E. coli* ER2538 cells, leading to a yield of a 8.3 × 10^7^ diverse library. Selection of specific sdAbs for the CD20 receptor was then performed using a subtractive cell phage display protocol as described in the material and methods section. This cell phage display screening was based on the previously Carlos Barbas studies, in which it is reported a novel whole-cell selection protocol with negative and positive selection steps (subtractive phage display) designed to remove antibodies reacting with common antigens^34^. Phage display conditions implemented herein are summarized in Fig. 5a. Briefly, four pannings were performed using a subtractive selection. To promote the elimination of non-specific antibodies, a negative selection was performed using the HEK 293 T cell line, which does not express the CD20. In addition, the CLBL-1 cell line was used for the positive selection due to its stable expression of CD20 receptor. Over the course of selection, stringency was incremented by increasing the number of washes in order to collect the phage clones with greater target affinity and specificity. As shown in Fig. 5b, the biopannings profile indicate that the pool of phages recovered from each round resulted in an enrichment of phage binders toward canine CD20 within the polyclonal pool. To further select the best anti-CD20 sdAb antibody lead candidates regarding their binding features and expression properties, the 4th panning phagemid DNA was cloned into a pT7-PL vector and transformed into *E. coli* strain BL21. A high throughput screening was then performed on a total of 528 clones aiming to select the antibodies in the sdAb format. The high throughput screening implemented allowed to select 12% of clones that presented a 4–5 higher-than-background signal against CLBL-1 protein extracts (Fig. 6a). Then, a total of 30 clones were randomly selected to be sequenced (data not shown). After sequencing analysis, 8 clones showed sequence variations in the CDR regions and were chosen for further binding characterizing studies against CLBL-1 and Raji protein extracts. As shown in Fig. 6b, all sdAbs bound to CLBL-1 and Raji extracts demonstrating that the selected clones specifically recognized the canine CD20 and presented cross-reactivity against human CD20.

#### Validation of canine CD20 targeting with sdAbs lead candidates

**Figure 7:**
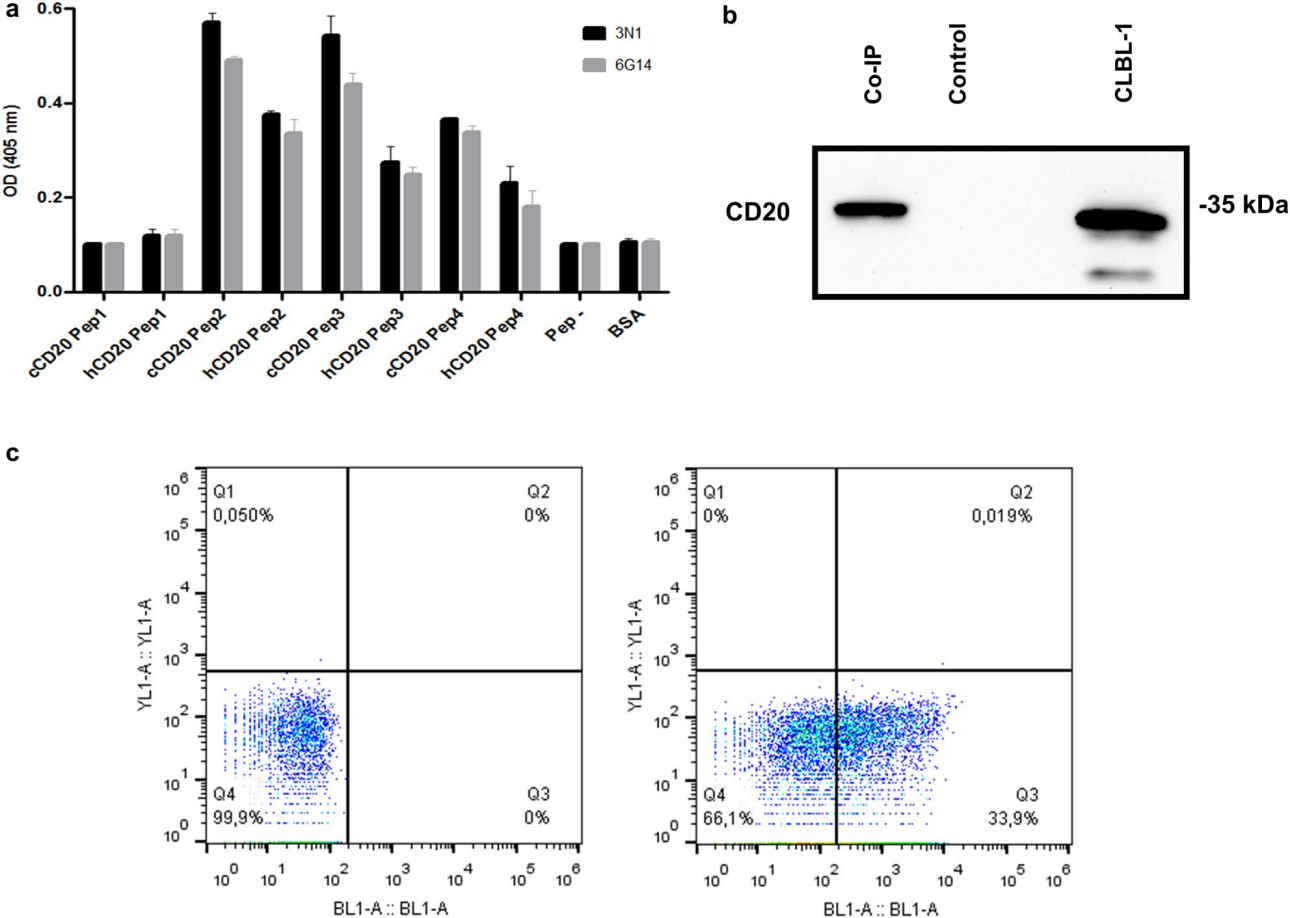
Target validation of the lead anti-CD20 sdAbs candidates. (**a**) Epitope mapping of the two lead candidates (3N1 and 6G14) selected based on their binding properties. For that purpose, sdAbs clones were tested for binding to a set of overlapping synthetic canine and human peptides (Supplementary Table S1) covering the two entire external domains (ED1 and ED2) by ELISA. Results were measured by optical density at 405 nm. Results are expressed as mean ± SEM. (**b**) Representative blots of CD20 WB analysis of Co-IP. To validate CD20 targeting of our sdAbs, 3N1 was selected as the most promising candidate based on its binding and expression properties. CD20 bound to 3N1 sdAb was pull downed using Dynabeads^®^ His-Tag Isolation & Pulldown and detected by WB analysis using anti-CD20 polyclonal antibody (CD20). Dynabeads^®^ His-Tag Isolation & Pulldown incubated with CLBL-1 cell extract without antibody were pull-down and used as a negative control. CLBL-1 cell line was used as a positive control. (**c**) Representative bivariate dot plots of FACS analysis of lead anti-CD20 sdAb candidate (3N1) binding activity. CLBL-1 cells were treated with 3 µM of 3N1 for 45 min and then sdAb binding to CLBL-1 cells were visualized by Alexafluor-488 labelling (dot plot on the right). Unstained CLBL-1 was used as a negative control (dot plot on the left).

To validate and characterize canine CD20 targeting by our lead sdAb candidates, two (3N1 and 6G14) out of the 8 clones were selected based on their high binding properties against CLBL-1 protein extract and expression yields. These two clones were expressed, and an epitope mapping was carried out as described in material and methods section. As showed in Fig. 7a, the two clones bound to the second larger extracellular domain of canine and human CD20. These results were concordant with results obtained from the rabbit sera epitope mapping, which also demonstrated a higher binding activity of the rabbit sera against the CD20 second extracellular domain.

Considering its promising binding and expression features, 3N1 sdAb was selected for further target characterization. First, to confirm the specificity of our anti-CD20 sdAbs binding towards canine CD20, a co-immunoprecipitation (co-IP) assay was performed. For that purpose, Dynabeads^®^ His-Tag isolation and pull-down (Thermo Fisher Scientific, Rockford, IL, USA) were incubated with our lead candidate 3N1 and CLBL-1 cell extracts as described in the material and methods section. As shown in Fig. 7b, the western blot analysis revealed that the co-IP study using 3N1 allowed to pull-down the canine CD20 receptor overexpressed on CLBL-1 cells.

Finally, to prove that 3N1 was also able to recognize canine CD20 in its native form, a flow cytometry study was conducted on the CLBL-1 cell line. Data shown in Fig. 7c demonstrated that 3N1 specifically bound to CLBL-1 cells. Live/dead reagent was used to exclude dead cells and the background noise was also evaluated in the control using the secondary antibody.

## Discussion

The inclusion of the cNHL model in the development of new anti-CD20 therapies may provide a powerful strategy for clinically relevant translation of improved therapeutics for treating relapsed and refractory disease of B cells malignancies. By gaining a better understanding of the complex immune interactions while addressing long-term efficacy and toxicity, anti-canine CD20 targeted immunotherapy research may serve as a predictive preclinical model, refining available therapeutic options and informing human clinical trials about novel approaches, while benefiting both species.

To the best of our knowledge, we conducted for the first time a comprehensive target characterization of the canine CD20. This data will be crucial to determine if tumor associated antigen specificity will guarantee high response rates and low toxicity^35^. Data generated from gene and protein expression studies on lymphoma and healthy canine cells revealed an overexpression of this receptor on cNHL cells, confirming the canine CD20 as a promising target for veterinary immunotherapies. Furthermore, when designing a passive immunotherapy similar to rituximab other clinical challenges must be considered, such as treatment resistance^36^. In fact, in human medicine only ~ 50% of patients with follicular lymphoma (FL) respond to initial treatment with single-agent rituximab^37^ and the majority of responders eventually become refractory to rituximab^38^. Thus, there is a pressing need to study treatment resistance including the factors responsible for innate rituximab sensitivity as well as the mechanisms responsible for acquired resistance. A proven predictor of innate rituximab sensitivity is CD20 surface expression in rituximab–naïve B cells^39^, demonstrating the importance of CD20 expression analysis before implementing an anti-CD20 immunotherapy. Lymphomas present heterogeneous levels of CD20 expression according to lymphoma types. For example, FL typically present higher levels of CD20 expression while CLL exhibit lower expression levels, which might justify the inferior efficacy of CLL cell lines to rituximab^20^. Acquired rituximab resistance following therapy exposure has likewise been associated with reduced levels of CD20, that are consistent with CD20 mRNA levels, indicating transcriptional control^40,41^. Therefore, by characterizing gene and protein CD20 expression levels on a cNHL naïve patient biobank, we expected to contribute for the research of predictive factor of response and possible mechanism of resistance that can overtime limit therapy responses, essential for the successful implementation of a canine anti-CD20 antibody-based therapy. Importantly, further studies enrolling larger sample size groups categorized according to cNHL subtypes are warranted in order to gain a better understanding of CD20 expression within and between lymphoma histological subtypes.

In addition, with our CD20 sequencing analysis we have identified 4 and 2 new amino acid sequence differences when compared with the previously CD20 sequences reported by Kano et al.^17^ and Beall and Elizabeth^27^, respectively. Importantly, three sequence differences were found on the two extracellular loops of canine CD20 (C77Y, L147F, I159M). Differences in amino acids 77, 147 and 159 residues might be relevant for the avidity of anti-CD20 canine mAbs and may impact its therapeutic and diagnostic use. Considering that the six amino acid differences were observed in all cNHL biobank, CLBL-1 cell line and healthy donors’ lymph nodes and PBMC samples, we believe that this might be the most predominant sequence of the canine CD20 and should be taken in consideration in the development of new therapeutic and diagnostic antibodies as well in clinical and therapeutic follow-up studies. Remarkably, studying CD20 expression and amino acid differences in dog breeds could also be relevant. In fact, the highly organized cross-generational pedigrees maintained by many breeders as well as the higher prevalence of lymphoma in certain dog breeds and the distribution of B-cell and T-cell lymphomas within specific breeds, represent a unique opportunity to study CD20 expression and amino acid differences and its impact in anti-CD20 therapies responses.

The human CD20 is a nonglycosylated 33 to 37-kDa integral membrane non-glycosylated phosphoprotein^42^. The 16-kb gene encoding human CD20, consists of 8 exons that have been mapped to chromosome 11 (11q12-q13) and belong to the MS4A (membrane spanning 4A) gene family^43^. The structure of human CD20 was recently reported^44^. Despite the prevailing notion that CD20 is a tetramer^45^, recent structural studies established CD20 as a compact dimeric double-barrel assembly, containing an intracellular termini and two extracellular loops of 9 and 46 residues spanning from amino acid 72 to 80 and from amino acid 142 to 188 (P178 to S188 extracelullar portion belongs to the transmembrane alpha helix), respectively^44^. The larger extracellular loop, mainly adjacent or amongst residues A170 and E174, comprises the known epitope recognized by rituximab and most other anti-CD20 mAbs^46^. Previously, collected data from antisera generated against peptides close to the amino and carboxyl termini and by proteolytic digestion of extracellular regions confirmed the membrane orientation and topology of CD20^47^. Rituximab binding was found to be imposed by both amino acid sequence and quaternary structure, justifying the heterogeneity in the fine specificity of CD20 mAbs^46^. Moreover, rituximab binding is inhibited by reduction and alkylation of CD20, which demonstrates that the epitope recognition is conformational^48^. Alanine-170 and Proline-172 were found to be critical determinants for extracellular CD20 epitopes, as their mutation in human CD20 abolished the binding of CD20 mAbs. The recent work developed by Rougé et al. confirmed that rituximab binds to the core epitope of CD20 ANPSE in amino acids 170–174^44^. However, this study in the human CD20 structure also revealed that rituximab binds to a second CD20 epitope formed by the smaller and larger loops and likely contributes substantially to rituximab affinity for CD20. Although the known epitope of rituximab and most anti-CD20 mAbs in human CD20 (170ANPSE174) located in the larger loop are relatively conserved in canine CD20 (including residues 170 and 172)^16,18^, one of the small loop residue left exposed by the shield of the larger loop and responsible by the rituximab binding to the secondary epitope I76 is not conserved in the dog. Moreover, the residue P178 located in the larger loop also responsible for the rituximab binding to the secondary epitope is also not conserved in the dog. Therefore, the lack of cross reactivity of canine CD20 to rituximab may be related to differences in the secondary epitope residues. Importantly, this lack of cross-reactivity of human anti-CD20 mAbs against other species, as mouse and dog, has limited the improvement of mechanisms and pharmacological characteristics of drugs targeting this receptor in naturally occurring animal models of B-cell lymphoma^19^. However, the investigation of the differences in the mechanism of action between human and canine anti-CD20 mAbs may provide insights into antibody development^49^.

Overall, data gathered under target characterization suggest that similarly to its human counterpart, canine CD20 has great potential to be used as a target for immunotherapy and immunodiagnostics, thus confirming and extending previous investigations. Despite the increasing investment in cNHL research, there is no anti-CD20 antibody-based therapy clinically available for cNHL to date. Within this context, in the present study we aimed to explore for the first time the potential of rabbit derived sdAbs to develop a new generation of anti-CD20 mAbs within the comparative oncology framework.

Several anti-CD20 mAbs derived from mouse have shown that can target canine CD20^19–21^. Ito et al*.* reported an anti-canine CD20 antibody (clone 6C8) that induced an ADCP response in canine B cells^19^. Jain et al*.* described an anti-canine CD20 antibody that possessed cross reaction with human CD20^21^. Rue et al*.* established an anti-canine CD20 antibody (clone 1E4) and developed a chimeric antibody for clinical use. In vitro cytotoxicity of this antibody via CDC and peripheral B cells in vivo depletion in healthy beagles were confirmed^20^. A recent work explored the potential of using rats as a host species to develop an anti-canine CD20 monoclonal antibody. Although this study identified a mAb capable of eliciting cell death of B cell lymphoma cell lines, this mAb did not showed CDC and ADCC activities. Hence, this mAb was improved into a canine/rat anti-CD20 chimeric and defucosylated, resulting in a potent CDC and ADCC inducer. This improved mAb version revealed tumoral growth inhibition in a murine model and a peripheral B cell depletion in healthy beagles^22^. Yet, the clinical efficacy of all these mAbs in dogs with B-cell lymphoma is still unknown. In addition, an anti-CD20 mAb (Blontuvetmab) has been approved in 2015 for canine B-cell lymphoma in the United States. Preliminary results obtained from a prospective randomized clinical trial indicated an improved median progression-free survival of dogs with B-cell lymphoma^50^. However, the results of this clinical trial were published in a conference abstract and the corresponding published peer-reviewed results are still not available.

To develop an effective CD20 targeted antibody-based therapy, we used a novel approach for the generation of recombinant anti-CD20 monoclonal antibodies against the coding sequence described herein, that binds both human and canine epitopes. For this purpose, we developed recombinant mAbs against canine CD20 through the construction of a sdAb library from a whole-cell immunized rabbit coupled with a phage display screening of the library on native-like receptor-expressed on cells. For decades, the rabbit immune repertoire has been used for the development of diagnostic polyclonal antibodies. More recently, the potential of rabbit antibodies has also been widely recognized as a promising source of therapeutic monoclonal antibodies^51^. Due to their B cell unique ontogeny, rabbits develop vastly distinctive antibody repertories with high diversity, affinity and specificity binders^52^. In addition, rabbits are evolutionarily distant from mice and rats and are able to generate antibodies against similar epitopes from different species, an attribute important for clinical translational^52^. Rabbit immunizations with HEK 293 T cells transfected with canine CD20 vector resulted in a specific and selective high-tittered antiserum against canine and human CD20. This strong and specific B-cell response allowed the construction of a high-quality sdAb gene library. sdAbs are presently the smallest functional antibody fragment, only consisting of an antibody V_H_ or V_L_. The small size of these fragments allows to improve tumor penetration and accessibility to targets not easily reached by IgGs. In addition to the reduced size, sdAbs possess complementary-determining regions (CDRs; antigen-binding regions) that are easily engineered into specific and high-affinity binders. Moreover, sdAbs also have unique characteristics including high stability, solubility, low immunogenicity and low manufacturing cost^29,53^.

To select a panel of rabbit derived CD20 targeting sdAbs, a phage display screening of the library on native-like receptor-expressed on cells was carried out to eventually potentiate the diversity of antibodies against this receptor^54^. A phage display screening using subtractive and positive pannings selected a specific pool of sdAbs-phages and high-throughput screening identified the best candidates. Next, an ELISA screening allowed the selection of the best sdAb candidates targeting both canine and human CD20. Finally, target validation and characterization studies allowed to confirm the specificity of the binding activity of these sdAbs against canine CD20 and to select a lead sdAb candidate (3N1) based on its promising binding and expression features. The availability of sdAbs against canine CD20, with cross-reactivity against human CD20, offers a platform for translational immunotherapy investigation. Moreover, the use of rabbit derived sdAbs allows to fully caninize the molecule and to promote ADCC responses after fusion to canine Fc constant regions as a traditional approach, or to develop more sophisticated recombinant molecules such as antibody-conjugates and bispecific antibodies.

In conclusion, our study provided new data characterizing canine CD20 as a promising target for immunotherapeutic strategies for veterinary settings, while contributing to comparative oncology. Future studies will determine canine CD20 expression value as a predictive factor of anti-CD20 immunotherapy. Moreover, we report herein novel sdAbs that recognize both canine and human CD20, that may become a useful tool for exploring the development of novel therapeutic alternatives and immunodiagnostics for comparative oncology. Future studies are necessary to test the therapeutic potential of these sdAbs in in vitro and in vivo experimental models.

## Methods

### Biological samples

#### Lymphoma group

Canine Multicentric Lymphoma Biobank Samples. Lymph node samples from a canine multicentric lymphoma biobank previously established by our group were used^24^. The cNHL biobank include blood and lymphoma affected lymph node samples from twenty two dogs diagnosed with naïve multicentric lymphoma followed at the oncology unit of the Veterinary Medicine Faculty—University of Lisbon (FMV/UL)’s—Teaching Hospital (Supplementary Table S1). The availability of this biobank allowed to access to multiple, properly preserved samples of lymph node sterile biopsies of well-characterized canine multicentric lymphoma cases. For diagnostic and staging purposes, all animals were submitted to a complete clinical evaluation. Complete blood count and biochemistry profile were performed, as well as abdominal and thoracic imaging exams. Histopathological evaluation of lymph nodes after node biopsy was performed. This histopathological evaluation included a morphologic examination, classification of lymphoma into grade subcategories and immunophenotyping to determine the immunophenotype present—B or T. Immunohistochemistry markers included CD3, CD20, CD79αcy and PAX-5. This clinical and laboratory examination allowed staging the dogs using the World Health Organization (WHO) system^55^.

#### Control group

Whole blood samples were collected from two canine healthy donors and processed for PBMC isolation and storage. All animals were submitted to clinical examination at FMV/UL’s—Teaching Hospital and their health status was screened through the execution of hematological and biochemistry profile and blood-borne parasites serology. Inclusion criteria included dogs aged between two and seven years, with a normal clinical examination and normal blood parameters. Exclusion criteria comprised dogs diagnosed with chronic diseases, such as heart disease, chronic kidney disease, endocrine disease or cancer, or whom have fall acutely ill and/or subjected to medications within the last 60 days. All sample collection was conducted with written informed pet owner consent in accordance with the principles and procedures outlined in the NIH Guide for the Care and Use of Animals and approved by the Animal Care and Use Committee of FMV/UL. All dogs that participated in this study were client-owned animals which joined the study voluntarily. All sampled animals stayed with their owners after sample collection. Due to ethical concerns raised by the collection of lymph nodes from healthy animals without medical indication, sterile biopsy lymph nodes samples were collected from a healthy control group of seven dogs housed at the animal facility of Faculty of Veterinary Medicine—Universiteit Utrecht. All sample collection was performed after donors were sacrificed by intravenous administration of 65 mg/kg sodium pentobarbital at the end of a non-related study. Lymph node samples were fined cut and stored at – 80 °C in RNAlater^®^ (Invitrogen, Life Technologies, Paisley, UK)^24^.

#### Cell lines and culture

The canine B-cell lymphoma cell line CLBL-1 was provided by Dr. Barbara Rütgen (University of Vienna, Austria). The human Burkitt's lymphoma Raji cell line and the human cell line HEK 293 T cell line (appropriated for ectopic expression of mammalian proteins) were obtained from the American Type Culture Collection (ATCC, Manassas, VA). CLBL-1 and Raji cell lines were maintained in RPMI-1640 medium (Gibco, Life Technologies, Paisley, UK) supplemented with 10% FCS (Gibco) and penicillin 100 U/ml/streptomycin 0.1 mg/mL (Gibco). HEK 293 T cell line was cultured in Dulbecco Modified Eagle Medium (DMEM) medium supplemented with 10% FCS (Gibco) and penicillin 100 U/mL/streptomycin 0.1 mg/mL (Gibco). All cell lines cultures were maintained at 37 °C in a humidified atmosphere of 5% CO_2_ (T75-tissue culture flasks, Greiner Bio-One, Kremsmünster, Austria).

### Characterization of canine CD20 expression in a canine multicentric lymphoma biobank

#### Relative quantification of canine CD20 expression by real-time qPCR

For total RNA extraction, lymph node samples stored in RNAlater^®^ from the canine multicentric lymphoma biobank^24^ and from the healthy control group were thawed and processed using RNeasy Mini Kit (Qiagen GmbH; Hilden, Germany), according to the manufacturer’s instruction^24^. To eliminate possible contaminant DNA, total RNA samples were subjected to DNAse treatment, using RNase-free DNase Set (Promega; Wood Hollow road, Madison, USA), following the manufacturer’s instructions. Thereafter, cDNA was synthesized using Transcriptor High Fidelity (Roche, Basel, Switzerland) following the manufacturer’s instructions and used as a template for Real-Time quantitative Polymerase Chain Reaction (qRT-PCR). Total RNA and cDNA purity were assessed by 1% agarose gel electrophoresis. The primers used are presented in Table 1. Despite the DNAse treatment, and to preclude genomic DNA amplification, primers covered putative exon–exon junctions. Optimization experiments and efficiency assessments for each amplification system were previously performed (data not shown). Primers were obtained from a commercial manufacturer (Metabion International AG, Germany). The mRNA transcription of the Ribosomal protein L27 gene (RPL27) had no significant statistical differences (p > 0.05) regarding cNHL and control groups, therefore this gene was considered a suitable housekeeping gene (Table 1). qRT-PCR was performed using the StepOne Plus realtime analyzer (Applied Biosystems, Foster City, CA, USA). The PCR assays comprised, in each reaction, 2 μL of each primer (final concentration of 100 nM), 2 μL of cDNA (1 ng), 4 μL of sterile water and 10 μL of SYBr (Applied Biosystems, Warrington, UK) in a total volume of 20 μL per reaction. Thermocycling conditions consisted of an initial denaturation of 10 min at 95 °C, followed by 40 cycles of amplification (95 °C for 15 s and annealing at 60 °C for 1 min). A final melting curve stage consisted of 95 °C for 15 s, 60 °C for 1 min followed by a ramp rate and heating of samples until 95 °C with a 0.3 °C/s ramp rate. The melting curves obtained were used to verify the specificity of each amplicon and finally PCR products were sequenced. The 2^− ΔΔCT^ method was used as described by Perkin-Elmer Applied Biosystems to assess relative mRNA expression quantification between lymphoma group and control group experiments^56^.

**Table 1:** Detailed primers and conditions used for RT-PCR and qRT-PCR assays used in CD20 gene studies.

Gene	Accession number	Sequence (5′–3′)	Amplicon size (bp)	Application
CD20	NM_001048028.1	FW-GGGCCCAGGCGGCCATGACAACACCCAGAAATTCA RV-CCTGGCCGGCCTGGCCAGGGATGCTGTCGTTTTCTATT	894	RT-PCR*
		FW-TGTCTATGCGCCCATCTGTATAA RV-TTTTCCTTTGACCAAACTCTTCCT	124	qRT-PCR*
RLP27	NM_001003102	FW-TCGTCAACAAGGATGTCTTCAGAG RV-TCTTGCCAGTCTTGTACCTCTCCT	96	qPCR*

#### Analysis of canine CD20 protein expression by immunoblotting

Lymph node samples stored in RNAlater^®^ from the canine multicentric lymphoma biobank^24^ and from the healthy control group were thawed and washed twice with PBS. Total protein was extracted using RIPA lysis buffer (25 mM TrisHCL pH 7.6, 150 mM NaCl, 1% NP-40, 1% sodium deoxycholate, 0.1% SDS) supplemented with fresh protease inhibitors (Roche) and phosphatase inhibitors cocktail (Sigma). Samples were quantified by the Bradford method. CD20 receptor expression was demonstrated by WB analysis. Total protein extract samples were separated by SDS-PAGE and transferred to nitrocellulose membranes. After blocking, proteins were incubated with anti-CD20 antibody (polyclonal, rabbit, 1:500 dilution, Thermo Fisher Scientific) and then with Peroxidase-AffiniPure anti-Rabbit IgG antibody (polyclonal, goat, 1:10,000 dilution, Jackson ImmunoResearch, PA, USA) as a secondary antibody. Proteins were visualized using Luminata Forte Western HRP (Merck Millipore, Darmstadt, Germany) and acquired using the ChemiDoc XRS + imaging system (Bio-Rad, California, USA). Beta-actin expression were used as protein loading control using an anti-β-actin antibody (AC-15 clone, mouse, 1:10,000 dilution, Sigma-Aldrich) as a primary antibody and an anti-Mouse IgG antibody (polyclonal, goat, 1:7500 dilution, Jackson ImmunoResearch) as a secondary antibody. Bioinformatic tools using Image Lab (Bio-rad) were used to quantify CD20 expression and demonstrate differences between samples.

#### Sequence analysis of the canine MS4A1 (CD20) gene

Total RNA was isolated from lymph nodes samples of the cNHL biobank and lymph node samples and PBMCs from the control group using Rneasy Mini Kit (Qiagen). To eliminate possible contaminant DNA, total RNA samples were subjected to DNAse treatment, using RNase-free DNase Set (Promega; Wood Hollow road, Madison, USA.), following the manufacturer’s instructions^24^. Thereafter, First-strand cDNA was synthesized using Transcriptor High Fidelity (Roche) following the manufacturer’s instructions and used as a template for RT-PCR using primers presented in Table 1, designed at the ends of reported sequence of canine CD20. Sequencing was performed by Eurofins Genomics (Ebersberg, Germany). Translation to amino acid sequences, multiple sequence alignment and phylogenetic analysis were performed using the “Vector NTI” software. Sequence alignments were processed using the “ALINE” software.

#### Statistical analysis

All data are expressed as mean ± standard error of mean (SEM). All statistical analyses were carried out using R-software. Normality test was performed using the Shapiro–Wilks test. The distribution of CD20 Ct values passed the normality test and lymphoma and control groups were therefore compared using Welch Two Sample t-test. The significance level was set at 5%.

### Generation of monoclonal antibodies against canine CD20

#### Cloning of canine CD20

The canine CD20 was cloned by RT-PCR from lymph node samples of a canine multicentric biobank case (patient 6) following the conditions previously mentioned above. PCR products were digested with *SfiI* (Roche) then purified and cloned into the FUGW vector, a gift from David Baltimore (Addgene plasmid # 14883; http://n2t.net/addgene:14883; RRID:Addgene_14883)^57^ at *SfiI* sites. Then, the ligated product was transformed into electrocompetent cells via thermal shock.

#### Transfection of canine CD20 into HEK 293 T cells

The HEK 293 T cells (ATCC) were transfected with plasmid DNA using the FuGENE^®^ HD (Roche), according to the manufacturer’s instructions. HEK 293 T cells were grown as monolayers in DMEM supplemented with 10% FCS and 3 μg of pFUGW vector CD20 was added. The HEK 293 T transfected cells were incubated for 48 h. Canine CD20 transient expression was confirmed by immunoblotting for canine CD20 expression (Thermo Fisher Scientific) and GFP staining with flow cytometry analysis. In parallel, a HEK 293T^hCD20^ cell line was simultaneously generated for cross-reactivity analysis purposes, using identical methodology.

#### Rabbit immunization

A New Zealand White rabbit was immunized with HEK 293 T cells transfected with canine CD20 vector. The injections were administrated subcutaneously at 2–3 week intervals during 4 months^58^.

#### Evaluation of rabbit immune response against canine CD20

The rabbit immune response developed against the canine and human CD20 was monitored by ELISA sera testing of the bleeds taken before and after each boost injection. Briefly, 60 × 10^3^ cells were blocked with PBS-BSA 1% (BSA, bovine serum albumin, Merck) for 30 min, washed with PBS and incubated with serial dilutions of the rabbit serum (from 1/500 to 1/60,000) for 1 h. Cells were then washed with PBS and secondary antibody goat-a anti-rabbit IgG-Fc specific HRP (Jackson ImmunoResearch) at 1:3000 in PBS-BSA 1% was added to each well and incubated for 1 h. Following incubation, ABTS substrate solution (Merck) was added, and optical density (OD) was measured with a microplate reader (Bio-rad) at 405 nm. Each serum was also analyzed for its binding profile against CD20 proteins extracts by WB. Briefly, CLBL-1, HEK 293 T cells with cCD20 vector, HEK 293 T cells with hCD20 vector and naïve HEK 293 T total protein extracts, obtained after RIPA cells lysis buffer (50 mM tris–HCl pH 7.4; 150 mM NaCl; 1% NP-40, 0.25% Na-deoxycholate), were separated by 12% SDS-PAGE and transferred into PVDF membranes as previously described. Then, WB was performed with each serum at 1/5000 dilution in PBS-BSA 1% followed by the anti-rabbit IgG-Fc specific HRP^59^.

In addition, an ELISA epitope mapping was performed. For that purpose, purified canine and human CD20 peptides (Supplementary Table S2) derived from the two external domains (ED 1 and ED2) were coated onto a CovaLink ELISA 96-well plates at 5 µg per well and allowed to bind for 1 h. The plate was washed three times with PBS 1×/Tween, blocked for 1 h with PBS 1×/BSA 3%, washed again and a solution of anti-CD20 rabbit serum were then added. The plate was incubated for 1 h, washed and bound mAbs were detected by incubating the plate an additional 1 h with Peroxidase-conjugated goat anti-rabbit antibody (Jackson Immune Research) as secondary antibody. ABTS reagent (Merck) was added, and plates were read at 405 nm after 5, 10, 30 and 60 min.

#### sdAbs immune library construction

Five days after the final boost, spleen and bone marrow were harvested separately for total RNA using TRI Reagent (Molecular Research Center, Cincinnati, OH). First-strand cDNA was synthesized using Transcriptor High Fidelity (Roche) following the manufacturer’s instructions. PCR was performed to amplify the V_L_ from the rabbit’s cDNA as previously described^29^. PCR products encoding a library of sdAbs were then gel purified, restriction digested with *Sfi*I and cloned into pcomb3×^60^. Subsequently, the ligated product was transformed into electrocompetent cells via electroporation and the library was tittered.

### Selection of sdAbs against canine CD20

#### Selection of anti-CD20 sdAbs by Phage Display

To display anti-CD20 sdAbs on M13 phage, sdAbs library was infected with M13 helper phage (10^12^ plaque-forming units/mL) during 1 h at 37 °C and grown overnight. Phages were precipitated, and titer was determined. The phage library displaying sdAbs was panned using a subtractive cell phage display protocol that included a negative selection on HEK 293 T cells followed by a positive selection on CLBL-1 cells. Four rounds of phage display panning were performed in order to select the best candidates. For each round, 5 × 10^6^ cells of HEK 293 T and CLBL-1 cell lines were washed with PBS 1× twice and blocked with PBS/BSA 1%, at room temperature (RT) for 15 min. Fresh phages were also blocked with PBS/BSA 1%. For the negative selection, 300 µL of phages were incubated with HEK 293 T cells for 15 min at 37 °C. Next, supernatant was recovered after cell centrifugation and unbound phages were incubated with CLBL-1 cells for 1.5 h at 37 °C for the positive selection. Next, CLBL-1 cells were washed with PBS 1× three times in the first round. In the second round, six wash steps were performed, and in the third and fourth rounds, cells were washed eight times. To recover bound phages, after the final wash step cells were resuspended in 200 µL of pre-warmed trypsin and incubated at 37 °C for 7 min. After trypsin dilution in 1.2 mL in PBS 1×, cells were centrifuged and the supernatant containing bound phages were tittered and stored at 4 °C. Following each panning, recovered phages were reamplified in bacteria and stored at – 80 °C.

#### In vitro binding and expression studies

A high throughput screening (Hamilton Automated System) was performed on a total of 528 clones aiming to select the mAbs in the sdAb format. Briefly, to express and select anti-CD20 sdAbs, phagemid DNA encoding selected anti-CD20 sdAbs from the phage display fourth round was cloned into pT7-PL (pT7-peptide leader) vector (Merck) and transformed into *E. coli* strain BL21. The colonies obtained were picked and incubated overnight at 30 °C on 100 µL of Super Broth (SB) medium containing Overnight Express™ Autoinduction System 1 (Novagen) and 100 µg/mL of ampicillin. Next day, 40 µL of BugBuster (Roche) containing anti-protease cocktail-EDTA free inhibitors (Roche) were added and incubated for 30 min at 4 °C. Next, the plates were centrifuged at 2000 rpm for 10 min and the supernatant was used to perform an ELISA assay. Three different parameters were analyzed: binding of antibodies to the antigen, expression level and unspecific binding. To evaluate the binding to the antigen (CD20), plates with the total CLBL-1 cell extract were used, washed with PBS and blocked with BSA 3% for 1 h at 37 °C. Then, plates were washed and the supernatant containing sdAb V_L_s was incubated for 1 h at 37 °C. Plates were washed and incubated with anti-HA-HRP antibody (Roche). After 1 h of incubation, plates were washed, ABTS (Roche) substrate solution was added and optical density at 405 nm was measured at different time points. To evaluate the level of expression, the same process was carried out, except for the antigen coating. To evaluate the unspecific binding, in turn of the antigen, BSA 3% was added. Rabbit serum and anti-CD20 antibody were used as positive controls. BL21 cell extracts were used as negative controls. A total of 30 clones were further tested by ELISA for their binding activity using CLBL-1 and Raji cell extract. Clones were sequenced by Eurofins Genomics using the pComb3x ATG primer. To translate to amino acid sequences and to evaluate homology, the Vector NTI Advance 10 software (Thermo Fisher Scientific) was used^58^.

#### Target validation assays

To evaluate the binding epitope of the selected anti-CD20 sdAbs, an epitope mapping assay was performed using the two sdAb candidates that demonstrated the most promising binding properties. For that purpose, the two best clones (3N1 and 6G14) were produced as described above and sdAbs were obtained by lysing the bacteria extract with BugBuster® (Roche) containing anti-protease cocktail-EDTA free inhibitors (Roche). The resultant supernatants were used for the epitope mapping, as previously described. In addition, to further validate and characterize our sdAbs binding properties against canine CD20, co-IP and flow cytometry assays were carried out. Based on its promising binding and expression properties, 3N1 sdAb was selected for these assays. To express and purify the 3N1 clone, a fresh colony of 3N1 clone was grown overnight at 37 °C in Super Broth (SB) medium containing 100 μg/mL of ampicillin. A 10 mL sample of cells was used to inoculate one liter of SB medium containing 100 μg/mL of ampicillin. Cells were grown at 37 °C until O.D.600 nm = 0.6, induced with 0.6 mM IPTG and growth was continued for 6 h at 19 °C. After induction, bacteria were harvested by centrifugation (4000×*g*, 4 °C, 15 min) and suspended in 50 mL equilibration buffer (20 mM NaH2PO4, 500 mM NaCl, 30 mM imidazole, and pH 7.4) supplemented with protease inhibitors (Merck). Cells were lysed by sonication. Centrifugation (14,000×*g*, 4 °C, 30 min) was used to remove cellular debris, and the supernatant was filtered through a 0.2 μm syringe filter. The sdAbs were then purified by immobilized metal affinity chromatography (IMAC), using HP Histrap columns and the AKTA Start system (Cytiva, Marlborough, Massachusetts, United States), using the C-terminal His6 of pT7-PL. After a washing step, elution of the sdAbs occurred by a linear imidazole gradient from 60 to 300 mM in elution buffer. The eluted fractions were pooled, desalted, and concentrated in PBS using 3K Amicon columns (Merck). Then, the sdAbs samples were loaded onto a HiPrep 16/60 Sephacryl S-100 HR gel filtration column (Cytiva) and pooled fractions were analyzed for protein purity by 15% SDS-PAGE followed by Coomassie blue staining and WB with HRP-conjugated anti-His antibody (Roche). The concentration of proteins was determined by measuring the absorbance at 280 nm in the Nanodrop 2000 (Thermo Fisher Scientific)^59^.

For co-IP, Dynabeads^®^ His-Tag Isolation & Pulldown were used following manufacturer’s instructions. 3N1 sdAbs (20 µg) prepared in 1× binding buffer were mixed with 50 µL of Dynabead^®^ magnetic beads and incubated for 10 min on a roller at RT. After discarding the supernatant, beads were washed 4 times with 1× binding buffer and incubated with CLBL-1 cell extract for 10 min on a roller at RT. After discarding the supernatant, beads were washed 4 times with 1× binding buffer and eluted with His-elution buffer. Samples were separated by SDS-PAGE and transferred to nitrocellulose membranes. Immunoblotting analysis of CD20 was conducted as previously described. For the flow cytometry analysis, CLBL-1 cells were washed twice in PBS, stained with LiveDead (Invitrogen) for 30 min RT. Cells were then washed twice with cold PBS-BSA 0.5% and incubated with 3 µM of 3N1 sdAb 45 min at 37 °C. Cells were then washed once with cold PBS-BSA 0.5%, fixed with PFA 2% for 10 min, washed twice with cold PBS-BSA 0.5% and incubated with anti-HA antibody (Roche) at 1:50 in PBS-BSA 0.5% for 30 min at 4 °C. Cells were then washed with cold PBS-BSA 0.5% 3 times and incubated with secondary antibody (Alexa Fluor^®^ 488 Goat Anti-Rat IgG) at 1:250 in PBS-BSA 0.5% for 30 min at 4 °C. Cells were washed with cold PBS-BSA 0.5% 3 times and submitted to FACS analysis (AttuneNXT). Unstained cells were used as negative control for voltage settings. LiveDead staining was used to exclude dead cells. For multiple-color sorts, single color controls were used for compensation settings. Data was analyzed by FlowJo software version 10 (FlowJo LLC).

## Supplementary Information

Supplementary Table S1.

Supplementary Table S2.
