# Supplementary material for: Characterization of the canine CD20 as a therapeutic target for comparative passive immunotherapy
Source: Sci Rep. 2022 Feb 17;12:2678. doi: 10.1038/s41598-022-06549-1 (PMC8854400; doi:10.1038/s41598-022-06549-1)
Supplement: Supplementary file 1 — Supplementary Table S1. [file 41598_2022_6549_MOESM1_ESM.pdf]

Table S1 - Canine multicentric lymphoma biobank clinical characteristics

Patient (n=22)	Identification	Age (Years)	Sex*	Breed	WHO Classification
1	B1	12	M	Schnauzer	DLBCL
2	T1	15	M	Mixed-breed	PTCL
3	B2	8	M	Labrador	DLBCL
4	N1	12	M	Golden Retriever	Blastic NK Lymphoma
5	B3	6	M	Rottweiller	DLBCL
6	B4	6	F	Basset Hound	DLBCL
7	B5	12	F	Pitbull	Follicular
8	B6	12	F	Husky	DLBCL
9	B7	8	M	Mixed-breed	DLBCL
10	B8	11	F	Mixed-breed	DLBCL
11	B9	9	F	Boxer	DLBCL
12	B10	7	M	Pointer	DLBCL
13	B11	17	F	Mixed-breed	DLBCL
14	B12	3	F	German Shepherd	DLBCL
15	B13	8	M	Mixed-breed	DLBCL
16	T2	9	M	Mixed-breed	T Zone Lymphoma
17	B14	4	M	Golden Retriever	DLBCL
18	T3	8	M	Beagle	PTCL
19	B15	5	M	Labrador	DLBCL
20	B16	9	F	Toy poodle	DLBCL
21	B17	11	F	Dobermann	DLBCL
22	B18	9	M	Afghan hound	DLBCL

*Sex M – Male, F – Female; PBMC – peripheral blood mononuclear cells; DLBCL – Diffuse Large B Cell Lymphoma; PTCL – Peripheral T Cell Lymphoma; NK – Natural Killer; [a] + tumor sample, - no tumor sample; [b] + PBMC sample, no PBMC sample; [c] + Serum sample, - no serum sample; [d] + submitted to CHOP protocol, - no CHOP protocol
