# Supplementary material for: Characterization of the canine CD20 as a therapeutic target for comparative passive immunotherapy
Source: Sci Rep. 2022 Feb 17;12:2678. doi: 10.1038/s41598-022-06549-1 (PMC8854400; doi:10.1038/s41598-022-06549-1)
Supplement: Supplementary file 2 — Supplementary Table S2. [file 41598_2022_6549_MOESM2_ESM.pdf]

Table S2 – Epitope mapping of canine and human CD20

Peptide ID	Amino acid position	Amino acid sequence
cCD20 Pep1 ED1	70-80 aa	LMIHTDVYAPI
cCD20 Pep2 ED2	145-189 aa	HFFKMENLNLIKAPMPYVDIHNCDPANPSEKNSLSIQYCGSIRSV
cCD20 Pep3 ED2	147-176 aa	FKMENLNLIKAPMPYVDIHNCDPANPSEKN
cCD20 Pep4 ED2	158-187 aa	PMPYVDIHNCDPANPSEKNSLSIQYCGSIR
hCD20 Pep1 ED1	70-80 aa	LLMIPAGIYAPI
hCD20 Pep2 ED2	145-189 aa	HFLKMESLNFIRAHTPYINIYNCEPANPSEKNSPSTQYCYSIQSL
hCD20 Pep3 ED2	147-176 aa	LKMESLNFIRAHTPYINIYNCEPANPSEKN
hCD20 Pep4 ED2	158-187 aa	HTPYINIYNCEPANPSEKNSPSTQYCYSIQ
Irrelevant Control Pep	1-20 aa	EQVDKLVSAGIRKVLFLDGI

cCD20 – Canine CD20; hCD20 – human CD20; ED1 – external domain 1; ED2 – external domain 2; aa- amino acid
